# Xiaozhi Yufa decoction ameliorates androgenetic alopecia through inhibition of MAPK signaling and regulation of lipid metabolism

**DOI:** 10.3389/fphar.2025.1724224

**Published:** 2025-11-27

**Authors:** Yiyu Guo, Jie Wu, Yu Lu, Jianping Qin, Lanlan Huang, Fengrui Zhang, Xiaomei Zhou, Dengke Yang, Jianzhou Ye

**Affiliations:** 1 The First Clinical Medical College, Nanjing University of Chinese Medicine, Nanjing, Jiangsu, China; 2 Kunming Municipal Hospital of Traditional Chinese Medicine, Kunming, Yunnan, China; 3 The First Affiliated Hospital of Yunnan University of Chinese Medicine, Kunming, Yunnan, China

**Keywords:** androgenetic alopecia, Xiaozhi Yufa decoction, traditional Chinese medicine, MAPK signaling, lipid metabolism, lipedematous alopecia

## Abstract

**Background:**

Androgenetic alopecia represents the most common form of progressive hair loss, with current treatments showing limitations in efficacy or tolerability. Xiaozhi Yufa decoction (XZYFD), a Traditional Chinese Medicine formulation composed of 13 herbal medicines, has shown clinical potential in treating hair loss.

**Methods:**

Network pharmacology analysis identified active compounds and potential targets of XZYFD, with molecular docking evaluating compound-target interactions. A testosterone propionate-induced mouse model was established to assess XZYFD’s therapeutic efficacy. Treatment effects were evaluated through hair regrowth assessment, histological examination, serum biochemical analysis, and molecular pathway investigation.

**Results:**

Network pharmacology identified 57 overlapping targets between XZYFD and androgenetic alopecia, with enrichment in MAPK signaling and lipid metabolism pathways. *In vivo* experiments demonstrated that XZYFD dose-dependently promoted hair regrowth and restored follicular morphology. Treatment significantly improved hormonal profiles, reduced serum lipid levels, and suppressed inflammatory markers. XZYFD effectively inhibited androgen metabolism and suppressed activation of MAPK signaling and SREBP-1-mediated lipid metabolism pathways, as confirmed through gene expression, protein analysis, and immunohistochemistry.

**Conclusion:**

XZYFD ameliorates androgenetic alopecia through simultaneous modulation of androgen metabolism, MAPK signaling, and SREBP-1-mediated lipid metabolism, with potential advantages for patients with metabolic dysfunction.

## 1 Introduction

Androgenetic alopecia represents the most common form of progressive hair loss, affecting a substantial proportion of both men and women worldwide. The condition typically manifests as gradual follicular miniaturization, leading to progressive thinning and eventual loss of terminal hair (Liu et al., 2025b). Epidemiological studies indicate that androgenetic alopecia affects approximately 50% of men by age 50 and a significant proportion of women during their lifetime (Tak et al., 2020; Ong et al., 2025). Beyond its physical manifestations, androgenetic alopecia exerts considerable psychological impact on affected individuals. Studies have demonstrated associations with reduced self-esteem, increased anxiety, and diminished quality of life (Huang et al., 2021). These psychosocial consequences underscore the clinical importance of developing effective therapeutic interventions for this condition.

The pathogenesis of androgenetic alopecia involves complex interactions between genetic predisposition, hormonal factors, and age-related changes (Xiao et al., 2025). Dihydrotestosterone (DHT) serves as the primary mediator of follicular miniaturization in genetically susceptible individuals. The enzyme 5α-reductase type 2 catalyzes the conversion of testosterone to DHT in hair follicles and sebaceous glands. DHT subsequently binds to androgen receptors in dermal papilla cells, triggering a cascade of events that shorten the anagen phase, prolong the telogen phase, and progressively reduce follicle size (Bellani et al., 2025). This process ultimately transforms terminal hairs into miniaturized vellus-like hairs, producing the clinical pattern of androgenetic alopecia. Understanding this hormonal mechanism has guided the development of current pharmacological treatments targeting androgen metabolism (Saceda-Corralo et al., 2023).

Current treatment approaches for androgenetic alopecia encompass both pharmacological interventions and non-pharmacological modalities including low-level laser therapy, platelet-rich plasma, and hair transplantation. Among pharmacological options, finasteride and minoxidil represent the most extensively studied and widely prescribed treatments, both demonstrating efficacy in clinical trials. Finasteride functions as a competitive inhibitor of 5α-reductase type 2, effectively reducing scalp and serum DHT levels. Clinical studies have shown that finasteride can halt hair loss progression and promote regrowth in a substantial proportion of treated men (Piraccini et al., 2022). However, concerns regarding potential adverse effects, including sexual dysfunction and mood alterations, have raised questions about long-term tolerability and patient adherence. Minoxidil, originally developed as an antihypertensive vasodilator, promotes hair growth through multiple mechanisms including enhancement of follicular blood flow, prolongation of the anagen phase, stimulation of vascular endothelial growth factor expression, and activation of potassium channels in follicular cells. While generally well-tolerated, minoxidil requires continuous application, and discontinuation typically results in loss of therapeutic gains (Olsen et al., 2025). These limitations of existing treatments highlight the need for alternative therapeutic approaches that may offer improved safety profiles or address additional pathological mechanisms.

Recent research has expanded understanding of androgenetic alopecia pathogenesis beyond simple androgenic mechanisms to encompass metabolic and inflammatory components. Studies have documented associations between androgenetic alopecia and metabolic abnormalities, including dyslipidemia and insulin resistance, suggesting that lipid metabolism dysregulation may contribute to disease pathology (Duan et al., 2025). The mitogen-activated protein kinase (MAPK) signaling pathways, including p38 MAPK and extracellular signal-regulated kinase 1/2 (ERK1/2), have emerged as important mediators of inflammatory responses and cellular stress in various dermatological conditions (Kanemaru et al., 2017). Evidence suggests that MAPK activation may contribute to follicular damage through promotion of inflammatory cytokine production and induction of apoptosis in follicular cells (Feng et al., 2025). Similarly, sterol regulatory element-binding protein-1 (SREBP-1) functions as a master regulator of lipogenic gene expression, controlling enzymes such as acetyl-CoA carboxylase 1 (ACC1) that govern fatty acid synthesis (Zhang et al., 2017). Dysregulation of SREBP-1-mediated lipogenesis could theoretically contribute to abnormal lipid accumulation in scalp tissue (Wang et al., 2020). Despite growing recognition of these metabolic and inflammatory components, therapeutic approaches targeting these pathways in androgenetic alopecia remain limited.

Traditional Chinese Medicine has employed herbal formulations for hair loss treatment for centuries, with clinical experience suggesting potential efficacy. Xiaozhi Yufa decoction (XZYFD) represents a multi-component formulation based on Traditional Chinese Medicine principles addressing both local and constitutional factors believed to contribute to hair loss. XZYFD was derived from two classical prescriptions, Pingwei San and Erzhi Wan, which have been traditionally used to address metabolic dysfunction and promote hair growth, respectively. Each constituent herb in the formulation has documented traditional use with established safety records within conventional dosage ranges. The formulation contains 13 herbal medicines selected for their purported effects on promoting blood circulation, resolving dampness, and nourishing hair follicles according to Traditional Chinese Medicine theory. The multi-component nature of such formulations suggests the possibility of simultaneous modulation of multiple pathological pathways, potentially offering advantages over single-target interventions. Network pharmacology has emerged as a valuable approach for investigating the complex mechanisms of herbal formulations, enabling prediction of active compounds, potential targets, and relevant signaling pathways (Nogales et al., 2022). This methodology aligns well with the multi-component, multi-target characteristics of Traditional Chinese Medicine and provides a framework for mechanistic investigation.

The present study aimed to investigate the therapeutic effects and underlying mechanisms of XZYFD in androgenetic alopecia, with particular focus on MAPK signaling and lipid metabolism pathways. We employed network pharmacology to predict potential active compounds and therapeutic targets, followed by experimental validation using a testosterone propionate-induced mouse model of androgenetic alopecia. The study assessed hair regrowth, histological changes, hormonal and metabolic parameters, inflammatory markers, and molecular pathway regulation to provide comprehensive characterization of XZYFD’s effects and mechanisms.

## 2 Materials and methods

### 2.1 Drugs and reagents

Testosterone propionate injection (25 mg/mL) was obtained from Hangzhou Animal Pharmaceutical Co., Ltd. (Hangzhou, China, batch number 230903). Finasteride tablets (1 mg per tablet) were purchased from Zhejiang Xianju Pharmaceutical Co., Ltd. (Hangzhou, China, approval number H20070112). For animal experiments, finasteride tablets were suspended in normal saline to achieve the desired concentration for oral gavage administration. Enzyme-linked immunosorbent assay (ELISA) kits were purchased from Nanjing Jiancheng Bioengineering Institute (Nanjing, China) with the following catalog numbers: testosterone (H090-1-1), DHT (H293-1–2), sex hormone-binding globulin (H657-1-1), total cholesterol (A111-1-1), triglycerides (A110-1-1), low-density lipoprotein cholesterol (A113-1-1), interleukin-1α (H001-1-1), interleukin-6 (H007-1-1), and interleukin-8 (H008-1-1). Primary antibodies against 5α-reductase 2 (sc-293232) were obtained from Santa Cruz Biotechnology (Dallas, TX, USA). Primary antibodies against phospho-p38 MAPK (AF4001), phospho-ERK1/2 (AF1015), phospho-SREBP-1 (AF3283), phospho-ACC1 (AF3421), and β-actin (AF7018) were purchased from Affinity Biosciences (Jiangsu, China).

### 2.2 Preparation of Xiaozhi Yufa decoction

XZYFD was composed of the following herbal medicines with their respective amounts. The formulation was derived from two classical Traditional Chinese Medicine prescriptions, Pingwei San and Erzhi Wan, with additional modifications based on clinical experience for treating hair loss with metabolic dysfunction. The dosage of each herbal component follows traditional clinical practice documented in classical Chinese medicine literature. *Pericarpium Citri Reticulatae* (Chenpi, dried tangerine peel, 15 g), *Poria sclerotium* (Fuling, poria sclerotium, 15 g), *Fructus Crataegi* (Shanzha, hawthorn fruit, 15 g), *Rhizoma Alismatis* (Zexie, alisma rhizome, 15 g), *Rhizoma Gastrodiae* (Tianma, gastrodia rhizome, 15 g), *Rhizoma Atractylodis Macrocephalae* (Baizhu, largehead atractylodes rhizome, 10 g), *Ligustri Lucidi Fructus* (Nvzhenzi, glossy privet fruit, 10 g), *Rhizoma Atractylodis* (Cangzhu, atractylodes rhizome, 20 g), *Ecliptae Herba* (Hanliancao, ecliptae herb, 20 g), *Platycladus orientalis Cacumen* (Cebaiye, oriental arborvitae leafy twig, 20 g), *Radix et Rhizoma Salviae Miltiorrhizae* (Danshen, salvia root, 20 g), *Cortex Magnoliae Officinalis* (Houpo, magnolia bark, 20 g), and *Radix et Rhizoma Glycyrrhizae* (Gancao, licorice root, 5 g) were included in the formulation. The total weight of the herbal mixture was 200 g per batch. The mixed herbal materials were subjected to aqueous extraction twice. In the first extraction, water was added at a ratio of 8:1 (volume to weight, 8 mL water per gram of herbal material, totaling 1600 mL for the 200 g mixture), brought to boil, and then decocted for 30 min. In the second extraction, water was added at a ratio of 6:1 (volume to weight, 6 mL water per gram of herbal material, totaling 1200 mL for the 200 g mixture), and the mixture was boiled and decocted for 20 min. The two decoctions were combined and filtered. The filtrate was concentrated under reduced pressure at 60 °C until a paste-like extract was obtained, followed by vacuum drying to constant weight. The final extract yield was approximately 20% based on the original crude drug weight.

### 2.3 Network pharmacology and molecular docking analysis

#### 2.3.1 Collection of XZYFD-related targets

Active compounds and their corresponding targets of XZYFD were retrieved from the Traditional Chinese Medicine Systems Pharmacology Database and Analysis Platform (TCMSP, https://www.tcmsp-e.com/). Compounds meeting the criteria of oral bioavailability (OB) ≥30% and drug-likeness (DL) ≥0.18 were selected as active ingredients. For herbs not found in TCMSP database, active compounds were retrieved from the HERB database (A high-throughput experiment- and reference-guided database of traditional Chinese medicine, http://herb.ac.cn/), and their corresponding SMILES structures were obtained from PubChem database (https://pubchem.ncbi.nlm.nih.gov/). The SMILES structures were then imported into SwissADME (http://www.swissadme.ch/) for pharmacokinetic prediction with the following criteria. Gastrointestinal absorption was set as “High” and at least two out of five drug-likeness rules (Lipinski, Ghose, Veber, Egan, and Muegge) were predicted as “Yes”. Subsequently, the SMILES structures of selected compounds were imported into Swiss Target Prediction database (http://www.swisstargetprediction.ch/), and targets with probability >0.1 were retained. The UniProt IDs of target proteins were converted to gene symbols using the ID mapping tool in UniProt database (https://www.uniprot.org/). After removing duplicates, the final XZYFD-related targets were obtained by merging data from TCMSP and HERB databases.

#### 2.3.2 Collection of androgenetic alopecia-related targets

Disease-related targets were retrieved from GeneCards (https://www.genecards.org/) and OMIM (https://www.omim.org/) databases using “Androgenetic alopecia” and “Lipedematous alopecia” as keywords. The retrieved targets from both databases were merged and deduplicated to obtain the final disease-related target set.

#### 2.3.3 Identification of common targets

The overlapping targets between XZYFD and androgenetic alopecia were identified using the online Venn diagram tool (https://bioinfogp.cnb.csic.es/tools/venny/). These common targets were considered as potential therapeutic targets of XZYFD for androgenetic alopecia treatment.

#### 2.3.4 Construction of protein-protein interaction network

The common targets were imported into the STRING database (https://cn.string-db.org/) with a minimum required interaction score ≥0.4 to construct the protein-protein interaction (PPI) network. The PPI network data were downloaded in TSV format and visualized using Cytoscape software (version 3.9.0) to construct the XZYFD-androgenetic alopecia multi-dimensional network.

#### 2.3.5 Gene Ontology and KEGG pathway enrichment analysis

Gene Ontology (GO) functional enrichment and Kyoto Encyclopedia of Genes and Genomes (KEGG) pathway enrichment analyses were performed using R software to explore the potential biological functions and major signaling pathways of XZYFD in treating androgenetic alopecia. Enrichment results with q value <0.05 were considered statistically significant and ranked by P-value in descending order. The top 20 KEGG pathways and significant GO biological functions were selected for visualization and analysis.

#### 2.3.6 Molecular docking

To validate the binding interactions between active compounds and target proteins identified through network pharmacology analysis, molecular docking studies were performed. Based on the degree values obtained from the protein-protein interaction network, the top three compounds with the highest degree values were selected for docking studies. These compounds were quercetin, 3′,4′,7-trihydroxyflavanone, and caulophyllogenin. The three-dimensional crystal structures of six key target proteins were retrieved from the RCSB Protein Data Bank (http://www.rcsb.org/) and saved in PDB format. These proteins included SRD5A2 (PDB ID: 7BW1), MAPK (PDB ID: 2Z8M), p38 MAPK (PDB ID: 1CM8), ERK (PDB ID: 5NHP), SREBP1 (PDB ID: 2J21), and ACC1 (PDB ID: 4ASI). The molecular structures of the three active compounds were downloaded from the PubChem database (https://pubchem.ncbi.nlm.nih.gov/) and saved in SDF format.

Protein structures were prepared using PyMOL software by removing water molecules and original ligands, and the processed structures were saved in PDB format. The Getbox Plugin in PyMOL was employed to determine the coordinates and dimensions of the binding pocket for each protein. Both the prepared protein structures and compound molecules were imported into AutoDock Tools 1.5.6 for format conversion, generating PDBQT files with appropriate charges and atom types assigned. Molecular docking calculations were subsequently performed using AutoDock Vina 1.1.2 with default parameters. The binding conformations with the lowest binding energies were selected as the optimal docking poses for each protein-ligand complex. Finally, the molecular docking results were visualized using PyMOL 2.6.0 to illustrate the binding modes and key interactions between compounds and target proteins.

### 2.4 Molecular dynamics simulation

To further evaluate the stability and dynamic behavior of the protein-ligand complex, molecular dynamics simulations were performed on the SRD5A2-caulophyllogenin complex, which exhibited the strongest binding affinity among all docking combinations. The initial structure was prepared from the optimal docking conformation obtained in section 2.3.6, and simulations were conducted using Gromacs 2022 software.

Force field parameters were obtained through the pdb2gmx tool in Gromacs and the AutoFF web server. The SRD5A2 protein was parameterized using the amber14sb force field, while caulophyllogenin was parameterized using the GAFF2 force field. The complex was solvated in a cubic TIP3P water box with a distance of 1 nm from the protein surface, and counter ions were added using the gmx genion tool to achieve electrical neutrality. Long-range electrostatic interactions were calculated using the Particle Mesh Ewald method with a cutoff distance of 1 nm. All bonds were constrained using the SHAKE algorithm, and the integration time step was set to 1 fs using the Verlet leapfrog algorithm. Prior to molecular dynamics simulation, the system underwent energy minimization consisting of 3000 steps of steepest descent optimization followed by 2000 steps of conjugate gradient optimization. The optimization procedure included three stages. First, energy minimization of water molecules with constrained solute was performed. Second, energy minimization with constrained counter ions was conducted. Finally, energy minimization of the entire system without constraints was carried out. The production simulation was conducted under NPT ensemble conditions at 310 K for 100 ns. During the simulation, root mean square deviation (RMSD), root mean square fluctuation (RMSF), hydrogen bonds (HBonds), and radius of gyration (Rg) were calculated using g_rmsd, g_rmsf, g_hbond, and g_gyrate tools respectively to assess the stability and conformational changes of the protein-ligand complex.

### 2.5 Model establishment and drug intervention

Male SPF-grade C57BL/6 mice (n = 30, 6–8 weeks old, 18–22 g body weight) were obtained from Beijing Sibeifu Biotechnology Co., Ltd. (SCXK (Beijing)2019–0010). Animals were housed under controlled conditions with temperature maintained at 20 °C–26 °C, relative humidity at 40%–70%, and a 12-h light/dark cycle. Standard commercial pellet diet (Irradiation-sterilized rodent diet, JXT standard; Jiangsu Xietong Pharmaceutical Bio-engineering Co., Ltd., Nanjing, China; Cat. No. XTI01JX-002) and water were available *ad libitum* throughout the experimental period. All mice underwent 1 week of acclimatization prior to experimentation. Animal procedures were approved by the institutional ethics committee (approval number: BST-PZ-MICE-20240228–01) and conducted in accordance with laboratory animal care guidelines.

Thirty mice were randomly divided into six groups (n = 5 per group) as follows. The control group, model group, XZYFD low-dose group (XZYFD-L), XZYFD medium-dose group (XZYFD-M), XZYFD high-dose group (XZYFD-H), and finasteride group were established. Twenty-four hours before model induction, a 2 cm × 3 cm area on the dorsal skin was shaved to bare skin level using an electric clipper along the spinal midline, ensuring complete removal of visible hair without causing skin damage. The androgenetic alopecia model was established by subcutaneous injection of testosterone propionate (0.075 mL per mouse) in the shaved dorsal area once daily for 14 consecutive days (Zhou et al., 2019). The control group received equivalent volumes of normal saline during the same period.

Following completion of the 14-day modeling period, drug interventions were initiated and continued for an additional 14 days. The control and model groups received normal saline by gavage once daily. XZYFD groups received XZYFD extract at doses of 4 g/kg (low), 8 g/kg (medium), and 16 g/kg (high), respectively, by gavage once daily. The finasteride group received finasteride suspension at 0.2 mg/kg by gavage once daily. Drug dosages were calculated based on interspecies dose conversion using body surface area normalization with a conversion factor of 12.3 (Nair et al., 2018). The human clinical dose of finasteride (1 mg/day, approximately 0.017 mg/kg for a 60 kg adult) was converted to the mouse equivalent dose of 0.2 mg/kg. Similarly, the traditional clinical dose of XZYFD (200 g crude drugs daily for a 60 kg adult, yielding 40 g extract at 20% yield rate, equivalent to 0.67 g/kg) was converted to mouse doses representing low, medium, and high dose groups respectively.

Hair regrowth progression was monitored and digitally photographed on days 0, 3, 7, 11, and 14 of the treatment period. Hair regrowth was qualitatively assessed by comparing hair coverage and density in the shaved area relative to pre-treatment baseline and control group levels. Assessment was performed by observers blinded to group assignments to minimize evaluation bias. At the study endpoint, mice were fasted for 12 h before being euthanized. Blood samples were collected for serum biochemical analysis, and dorsal skin tissues were harvested for histological examination and molecular biological analyses.

### 2.6 Histological examination

At the study endpoint, dorsal skin tissues from the shaved areas were immediately excised and fixed in 4% paraformaldehyde solution for 24 h at 4 °C. Following fixation, tissues were dehydrated through graded ethanol series, cleared in xylene, and embedded in paraffin blocks using standard histological procedures. Serial sections of 4 μm thickness were cut using a rotary microtome and mounted on glass slides. Sections were deparaffinized in xylene, rehydrated through descending grades of ethanol, and stained with hematoxylin and eosin according to standard protocols. Briefly, sections were stained with hematoxylin for 5 min, differentiated in acid alcohol, blued in alkaline water, and counterstained with eosin for 2 min. After dehydration and clearing, sections were mounted with neutral balsam and coverslipped. Histological examination was performed using a light microscope at ×100 magnification to observe hair follicle morphology, dermal structure, and general tissue architecture following standard protocols (Yan et al., 2024). Representative photomicrographs were captured using a digital imaging system for documentation.

### 2.7 Serum biochemical analysis

Blood samples were collected from the orbital venous plexus of mice at the study endpoint after 12 h of fasting. Blood was allowed to clot at room temperature for 30 min, then centrifuged at 3,000 × g for 10 min at 4 °C to separate serum. Serum levels of nine biochemical parameters were measured using ELISA kits according to the manufacturers' instructions. The analyzed parameters included testosterone, DHT, SHBG, TC, TG, LDL-C, IL-1α, IL-6, and IL-8. Optical density values were measured at 450 nm using a microplate reader, and concentrations were calculated from standard curves generated with known concentrations of each analyte.

### 2.8 RNA extraction and quantitative real-time PCR

Dorsal skin tissues were collected and immediately snap-frozen in liquid nitrogen, then stored at −80 °C until RNA extraction. Approximately 100 mg of dorsal skin tissue was used for each RNA extraction. Total RNA was extracted using TRIzol reagent according to the manufacturer’s protocol. RNA concentration and purity were determined by measuring absorbance at 260 nm and 280 nm using a NanoDrop spectrophotometer. Only samples with A260/A280 ratios between 1.8 and 2.0 were used for subsequent analysis. First-strand complementary DNA (cDNA) was synthesized from 1 μg of total RNA using a reverse transcription kit according to the manufacturer’s instructions. Quantitative real-time PCR (qRT-PCR) was performed using SYBR Green Master Mix on a real-time PCR system. The reaction conditions included initial denaturation at 95 °C for 10 min, followed by 40 cycles of denaturation at 95 °C for 15 s, annealing at 60 °C for 30 s, and extension at 72 °C for 30 s. The target genes analyzed included 5α-reductase 2, p38 MAPK, ERK1/2, SREBP-1, and ACC1, with GAPDH serving as the internal reference gene. All primers were synthesized by Sangon Biotech (Shanghai) Co., Ltd. (Shanghai, China). Primer sequences, amplicon sizes, and GenBank accession numbers are listed in Table 1. All reactions were performed in triplicate, and relative gene expression levels were calculated using the 2^−ΔΔCt^ method.

**TABLE 1:** Primer sequences used for RT-q PCR.

Gene	Forward (5′–3′)	Reverse (5′–3′)	Amplicon size (bp)	GenBank accession
5α-reductase 2	GCA​TCC​TTG​AGA​AAG​GGG​TA	CTC​TGC​TGG​CAC​TGA​ACC​T	112	NM_053188
p38 MAPK	GGA​TAT​TTG​GTC​CGT​GGG​CT	GTT​TCT​TGC​CTC​ATG​GCT​TGG	132	NM_011951
ERK1/2	CCC​AAA​TGC​TGA​CTC​CAA​AGC	TTG​AAT​GGC​GCT​TCA​GCA​AT	138	NM_011949
SREBP-1	CGA​AGT​GGT​GGA​GAC​GCT​TA	ACC​TGG​CTA​TCC​TCA​AAG​GC	142	NM_011480
ACC1	CAG​CAT​CTC​TAA​CTT​CCT​TCA​CTC​C	ACA​CGA​GCC​ATT​CAT​TAT​CAC​TAC​G	158	NM_133360
GAPDH	AGG​TCG​GTG​TGA​ACG​GAT​TTG	TGT​AGA​CCA​TGT​AGT​TGA​GGT​CA	123	NM_001289726

### 2.9 Protein extraction and Western blot analysis

Dorsal skin tissues were collected and immediately snap-frozen in liquid nitrogen, then stored at −80 °C until protein extraction. Tissues were homogenized in RIPA lysis buffer containing protease and phosphatase inhibitors using a tissue homogenizer. The homogenates were centrifuged at 12,000 × g for 15 min at 4 °C, and the supernatants were collected. Protein concentrations were determined using the BCA protein assay kit according to the manufacturer’s instructions. Equal amounts of protein (30 μg) were separated by SDS-PAGE and transferred to PVDF membranes. Membranes were blocked with 5% non-fat milk in TBST for 1 h at room temperature, then incubated with primary antibodies overnight at 4 °C. The target proteins analyzed included 5α-reductase 2, p-p38 MAPK, p-ERK1/2, p-SREBP-1, and p-ACC1, with β-actin serving as the loading control. After washing with TBST, membranes were incubated with HRP-conjugated secondary antibodies for 1 h at room temperature. Protein bands were visualized using ECL detection reagent and captured using a chemiluminescence imaging system. Band intensities were quantified using image analysis software and normalized to β-actin levels.

### 2.10 Immunohistochemical staining

Immunohistochemical staining was performed following established protocols (Charoensuksira et al., 2024). Paraffin-embedded dorsal skin tissue sections of 4 μm thickness were deparaffinized in xylene and rehydrated through descending grades of ethanol. Antigen retrieval was performed by heating sections in citrate buffer (pH 6.0) using a microwave for 15 min. Sections were cooled to room temperature and washed with phosphate-buffered saline (PBS). Endogenous peroxidase activity was blocked with 3% hydrogen peroxide for 10 min, followed by blocking with 5% normal goat serum for 30 min at room temperature. Sections were then incubated with primary antibodies against p-p38 MAPK, p-ERK1/2, p-SREBP-1, and p-ACC1 overnight at 4 °C. After washing with PBS, sections were incubated with HRP-conjugated secondary antibodies for 30 min at room temperature. Immunoreactive signals were visualized using DAB substrate solution until appropriate staining intensity was achieved. Sections were counterstained with hematoxylin, dehydrated through graded alcohols, cleared in xylene, and mounted with neutral balsam. Representative photomicrographs were captured using a light microscope with digital imaging system.

### 2.11 Statistical analysis

All experimental data were expressed as mean ± standard deviation (SD) and analyzed using GraphPad Prism software (version 8.0). Prior to ANOVA analysis, data normality was assessed using the Shapiro-Wilk test, and homogeneity of variance was evaluated using Levene’s test. All datasets satisfied the assumptions for ANOVA analysis (*p* > 0.05 for both normality and homogeneity tests). The independence of observations was ensured by random allocation of animals to experimental groups and independent measurement procedures. Statistical comparisons among multiple groups were performed using one-way analysis of variance (ANOVA) followed by Tukey’s *post hoc* test for pairwise comparisons. A probability value of *P* < 0.05 was considered statistically significant. Significance levels were indicated as *P < 0.05, **P < 0.01, ***P < 0.001, and ****P < 0.0001. All experiments were performed with appropriate biological replicates as specified in each experimental section.

## 3 Results

### 3.1 Network analysis reveals MAPK and lipid metabolism as key therapeutic pathways

**FIGURE 1:**
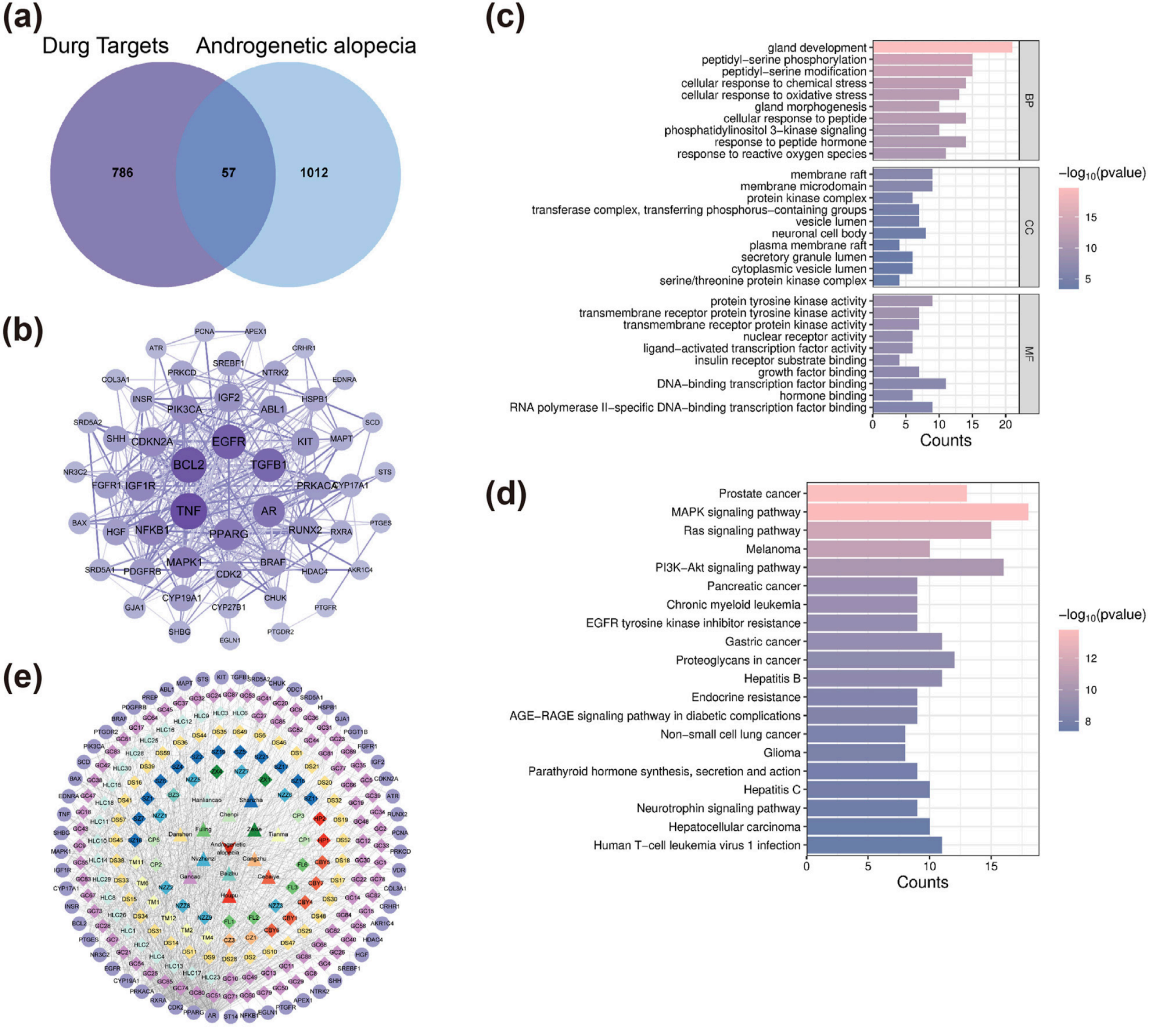
Network pharmacology analysis identifies key targets and pathways of XZYFD in treating androgenetic alopecia. **(a)** Venn diagram showing the intersection of XZYFD-related targets and androgenetic alopecia-related targets. **(b)** Protein-protein interaction network of 57 overlapping targets constructed using the STRING database and visualized by Cytoscape software. Node size represents degree value, and edge thickness indicates interaction confidence. **(c)** Gene Ontology enrichment analysis showing the top 20 biological processes, cellular components, and molecular functions associated with the overlapping targets. **(d)** KEGG pathway enrichment analysis displaying the top 20 significantly enriched signaling pathways. **(e)** Compound-target-pathway network illustrating the multi-component and multi-target characteristics of XZYFD. Polygonal nodes represent pathways, circular nodes represent targets, and diamond nodes represent active compounds.

A total of 309 active compounds and 843 corresponding targets of XZYFD were retrieved from the TCMSP and HERB databases. The herbal composition analysis identified varying numbers of active compounds across the 13 constituent herbs. *Pericarpium Citri Reticulatae* contained 5 active compounds, *Poria* contained 15 compounds, *Fructus Crataegi* contained 34 compounds, *Rhizoma Alismatis* contained 10 compounds, *Rhizoma Gastrodiae* contained 24 compounds, *Rhizoma Atractylodis Macrocephalae* contained 7 compounds, *Ligustri Lucidi Fructus* contained 13 compounds, *Rhizoma Atractylodis* contained 9 compounds, *Ecliptae Herba* contained 40 compounds, *Platycladus orientalis* contained 7 compounds, *Radix et Rhizoma Salviae Miltiorrhizae* contained 65 compounds, *Cortex Magnoliae Officinalis* contained 2 compounds, and *Radix et Rhizoma Glycyrrhizae* contained 92 compounds. Disease-related target screening from the GeneCards and OMIM databases identified 1069 targets associated with androgenetic alopecia. The intersection analysis between XZYFD targets and androgenetic alopecia-related targets revealed 57 overlapping targets, representing potential therapeutic targets for XZYFD in treating androgenetic alopecia (Figure 1a).

The drug-compound-target-disease network was constructed using Cytoscape software (version 3.9.0) to visualize the relationships between XZYFD components and androgenetic alopecia targets. The network comprised 251 nodes, including 180 active compound nodes, 57 target nodes, 13 herbal medicine nodes, and 1 disease node, connected by 981 edges. Topological analysis using the Network Analyzer plugin revealed a mean degree of 7.817, network heterogeneity of 1.677, network density of 0.031, and network centralization of 0.416. Nodes with higher degree values were considered core components in the network. The top-ranked active compounds by degree value included quercetin (GC88/CBY7/NZZ9/HLC25, degree equal to 21), 3′,4′,7-trihydroxyflavanone (HLC1, degree equal to 14), caulophyllogenin (HLC10, degree equal to 14), esculentic acid (HLC16, degree equal to 13), and ethoxysanguinarine (TM6, degree equal to 13) (Table 2). These findings demonstrated that XZYFD exerts therapeutic effects on androgenetic alopecia through multiple compounds acting on multiple targets simultaneously.

**TABLE 2:** Core active ingredients of XZYFD.

Name	Molecule_name	Degree	BetweennessCentrality	ClosenessCentrality
GC88/CBY7/NZZ9/HLC25	Quercetin	21	0.023359139	0.446428571
HLC1	3′,4′,7-trihydroxyflavanone	14	0.014068359	0.411184211
HLC10	Caulophyllogenin	14	0.011393739	0.409836066
HLC16	Esculentic acid	13	0.009375838	0.408496732
TM6	Ethoxysanguinarine	13	0.011916518	0.383435583
SZ19	Linolenic acid	13	0.009729024	0.40192926
HLC3	5-(3-buten-1-ynyl)-2,2′-bithienyl-5′-methyl-acetate	12	0.002038596	0.299043062
HLC12	Echinocystic acid	12	0.008533789	0.407166124
HLC13	Ecliptasaponin a_qt	12	0.008533789	0.407166124
HLC14	Ecliptasaponin d_qt	12	0.008533789	0.407166124
HLC15	Ecliptasaponin_qt	12	0.008533789	0.407166124
HLC6	Alpha-terthienyl methyl acetate	11	0.001850365	0.301204819
SZ11	Chlorogenin	11	0.007033597	0.385802469
SZ17	Ethylnotopterol	11	0.006665891	0.37993921
SZ21	Proscillaridin a	11	0.014222406	0.407166124
DS9/NZZ8/HLC18	Luteolin	11	0.009449292	0.420875421
HLC29	Yakuchinone b	10	0.005927977	0.378787879
TM12	Suchilactone	10	0.007135586	0.366568915
CZ1	Wogonin	10	0.011575195	0.420875421

Protein-protein interaction network analysis was performed to elucidate the mechanisms underlying XZYFD treatment of androgenetic alopecia. The 57 overlapping targets were imported into the STRING database, generating a PPI network containing 53 proteins and 357 interaction edges (Figure 1b). Visualization and analysis in Cytoscape software identified the top 5 hub proteins based on degree values. TNF exhibited the highest degree value of 36, followed by BCL2 (degree equal to 34), EGFR (degree equal to 32), TGFB1 (degree equal to 30), and AR (degree equal to 27). These hub proteins likely play important roles in the pathogenesis and treatment of androgenetic alopecia.

Gene Ontology enrichment analysis yielded 1942 entries for the 57 intersection targets. Biological process analysis identified 1839 entries, with the most significantly enriched processes including gland development, peptidyl-serine phosphorylation, and peptidyl-serine modification (Figure 1c). Molecular function analysis revealed 78 entries, predominantly related to protein tyrosine kinase activity, transmembrane receptor protein tyrosine kinase activity, and transmembrane receptor protein kinase activity. Cellular component analysis produced 25 entries, mainly associated with membrane raft, membrane microdomain, and protein kinase complex. These results indicated that XZYFD may regulate multiple biological processes including inflammatory responses and cell cycle progression in treating androgenetic alopecia.

KEGG pathway enrichment analysis identified 148 significantly enriched signaling pathways (q value less than 0.05) for the 57 intersection targets (Figure 1d). The most significantly enriched pathways included prostate cancer, MAPK signaling pathway, Ras signaling pathway, melanoma, PI3K-Akt signaling pathway, pancreatic cancer, chronic myeloid leukemia, EGFR tyrosine kinase inhibitor resistance, gastric cancer, and proteoglycans in cancer. The compound-target-pathway network was constructed and comprised 230 nodes and 911 connections. Topological analysis revealed a mean degree of 7.922, network heterogeneity of 1.623, network density of 0.035, and network centralization of 0.458 (Figure 1e). These findings demonstrated that XZYFD active compounds interact with multiple targets across different pathways, exhibiting multi-component, multi-target, and multi-pathway characteristics in treating androgenetic alopecia.

### 3.2 Molecular docking validates compound-target binding interactions

**FIGURE 2:**
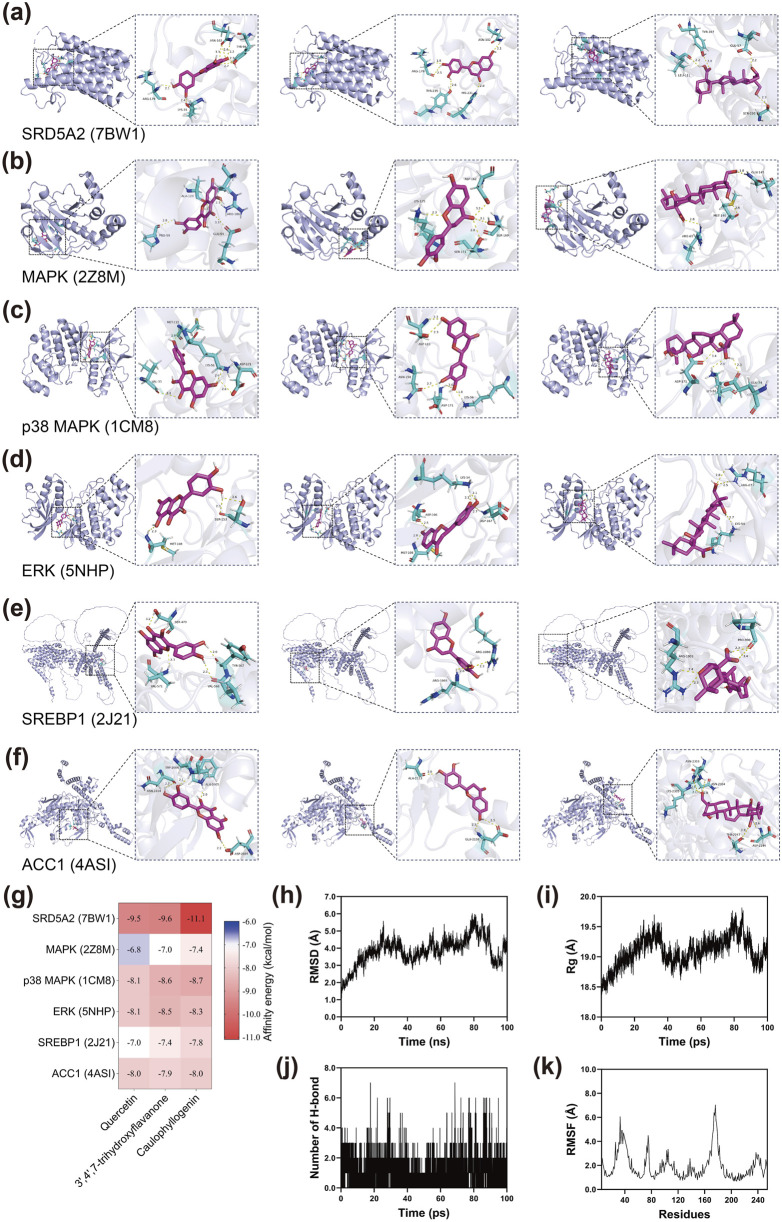
Molecular docking and dynamics simulation of active compounds from XZYFD with key target proteins. **(a–f)** Molecular docking poses showing the binding interactions between three active compounds (quercetin, 3′,4′,7-trihydroxyflavanone, and caulophyllogenin) and six target proteins (SRD5A2, MAPK, p38 MAPK, ERK, SREBP1, and ACC1). Protein structures are shown in ribbon representation, and compounds are displayed in stick format with key interacting residues highlighted. **(g)** Heatmap displaying the binding affinity values between compounds and target proteins. **(h)** Root mean square deviation of the SRD5A2-caulophyllogenin complex during 100 ns molecular dynamics simulation. **(i)** Radius of gyration of the protein structure throughout the simulation period. **(j)** Number of hydrogen bonds formed between SRD5A2 and caulophyllogenin over time. **(k)** Root mean square fluctuation values for individual residues of SRD5A2, indicating the flexibility of different protein regions.

Based on the degree values obtained from network pharmacology analysis, the top three active compounds (quercetin, 3′,4′,7-trihydroxyflavanone, and caulophyllogenin) were selected for molecular docking studies with six key target proteins including SRD5A2, MAPK, p38 MAPK, ERK, SREBP1, and ACC1. The docking results revealed varying binding affinities across different compound-target combinations (Figures 2a–g). SRD5A2 exhibited the strongest binding affinity with all three compounds, particularly with caulophyllogenin, which showed the most favorable interaction among all docking combinations. The MAPK pathway-related proteins demonstrated moderate to strong binding affinities with the three compounds. p38 MAPK and ERK both displayed favorable binding interactions, with caulophyllogenin and 3′,4′,7-trihydroxyflavanone showing slightly stronger affinities than quercetin. Lipid metabolism-related proteins ACC1 and SREBP1 also demonstrated favorable binding interactions with all three compounds. These binding energy values indicated that the active compounds could effectively bind to the predicted target proteins, supporting the network pharmacology predictions.

Molecular dynamics simulations were performed on the SRD5A2-caulophyllogenin complex to evaluate binding stability over 100 ns. The root mean square deviation analysis showed that the complex underwent structural adjustments during the simulation period, with RMSD values fluctuating between 3.0 and 6.0 Å throughout most of the trajectory (Figure 2h). Notable variations were observed between 20 and 90 ns, with peak values occurring around 80–90 ns before declining in the final phase. The radius of gyration analysis revealed moderate fluctuations in protein compactness, with Rg values varying within a range of approximately 1.2 Å (Figure 2i). Hydrogen bond analysis demonstrated dynamic interactions between SRD5A2 and caulophyllogenin, with the number of hydrogen bonds fluctuating between 0 and 7 throughout the simulation (Figure 2j). The complex maintained between 1 and 4 hydrogen bonds for most of the simulation period, indicating continuous molecular interactions despite variations in bond numbers. The root mean square fluctuation analysis identified regions of varying flexibility within the SRD5A2 structure (Figure 2k). Most residues exhibited relatively low RMSF values, indicating rigid structural segments. However, several regions showed elevated flexibility, with peaks observed around residues 40, 80, 160, and 220, suggesting the presence of loop regions or structurally flexible segments. These molecular dynamics results provided insights into the dynamic behavior of the SRD5A2-caulophyllogenin complex at the atomic level.

### 3.3 XZYFD restores hair growth and follicle morphology

**FIGURE 3:**
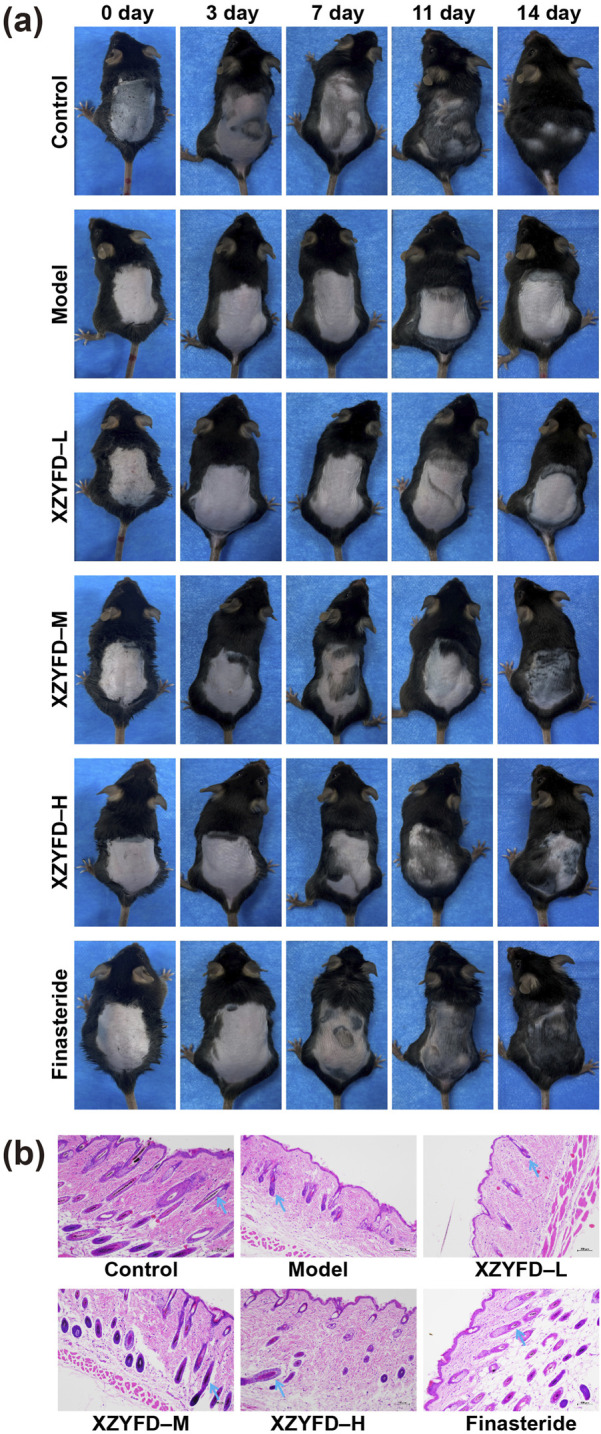
XZYFD restores hair growth and follicle morphology in androgenetic alopecia mice. **(a)** Representative photographs showing hair regrowth progression on the dorsal skin of mice at days 0, 3, 7, 11, and 14 of treatment. **(b)** Hematoxylin and eosin staining of dorsal skin tissue sections from different treatment groups. Scale bar represents 100 μm. Blue arrows indicate hair follicles.

Visual observation revealed distinct hair regrowth patterns among experimental groups during the 14-day treatment period (Figure 3a). The control group maintained normal hair growth throughout the observation period, achieving complete coverage of the shaved area by day 14. In contrast, the model group exhibited minimal hair regrowth, with the treated area remaining largely bare at the final time point. XZYFD treatment promoted hair regrowth in a dose-dependent manner. The low-dose group showed modest improvement with gradual hair emergence beginning around day 7. The medium-dose group demonstrated more pronounced regrowth, with visible hair appearing earlier and achieving substantial coverage by day 14. The high-dose XZYFD group displayed the most robust response, with hair emergence evident by day 3 and considerable coverage approaching control group levels by day 14. The finasteride group exhibited effective hair regrowth comparable to the high-dose XZYFD group, with substantial coverage achieved by the end of treatment.

Histological examination revealed marked differences in hair follicle morphology and dermal structure among treatment groups (Figure 3b). The control group exhibited normal architecture with well-developed hair follicles displaying typical anagen-phase characteristics. The model group showed significant alterations including atrophic and miniaturized follicles, increased dermal thickness, and expanded subcutaneous adipose tissue characteristic of lipedematous alopecia. XZYFD treatment resulted in progressive improvement in follicle morphology across dosage groups. The low-dose group demonstrated partial restoration with increased follicle size compared to the model group. The medium-dose group exhibited more substantial improvement with better organized follicular structures and increased follicle depth. The high-dose XZYFD group displayed the most pronounced restoration, with follicles showing normal morphology, deep extension into subcutaneous tissue, and reduced lipedematous changes. The finasteride group exhibited similar histological improvements to the high-dose XZYFD group, with restored follicle size and improved dermal organization.

### 3.4 XZYFD modulates serum biochemical parameters

**FIGURE 4:**
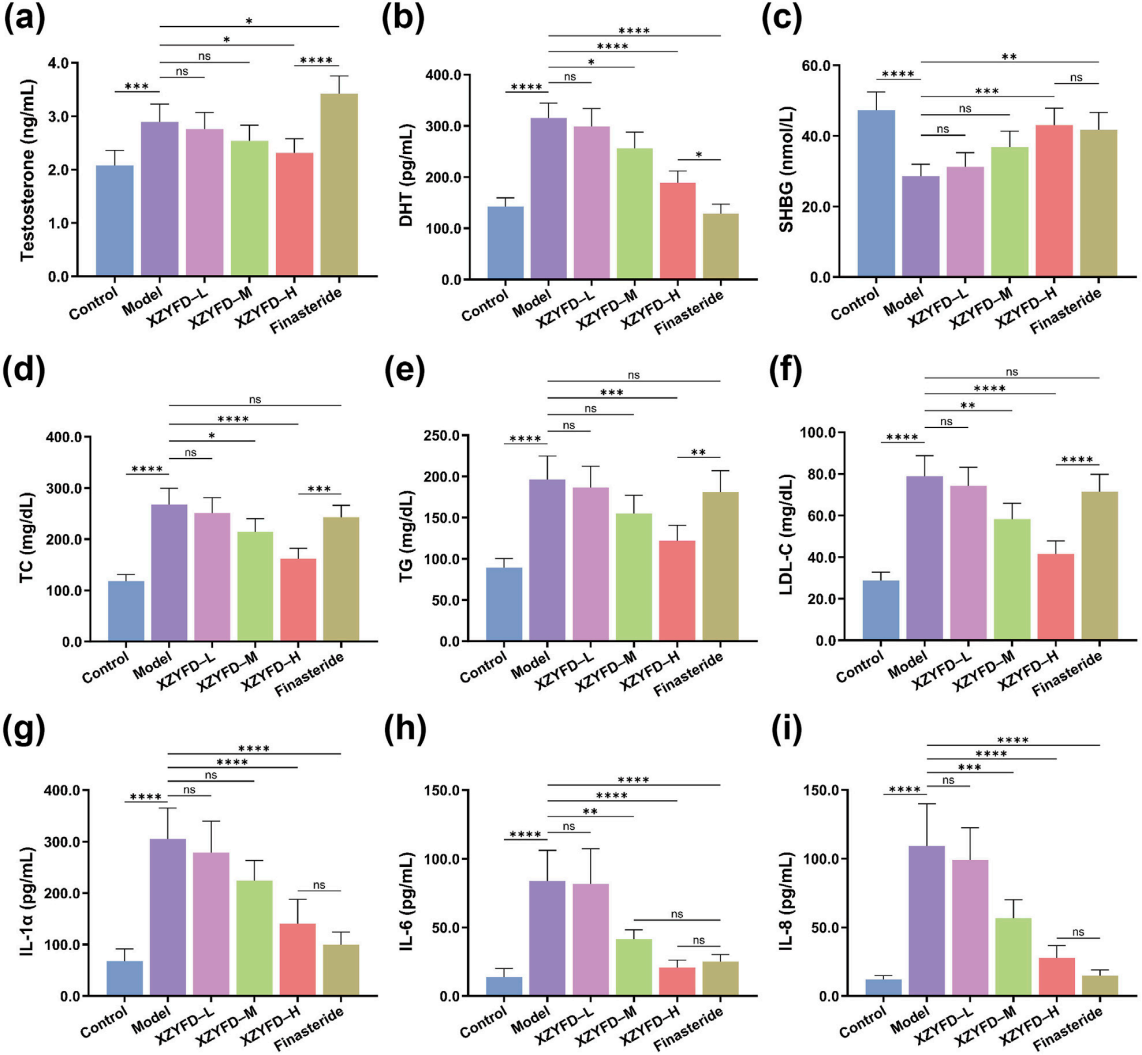
XZYFD modulates serum biochemical parameters in androgenetic alopecia mice. Serum levels of **(a)** testosterone, **(b)** dihydrotestosterone, **(c)** sex hormone-binding globulin, **(d)** total cholesterol, **(e)** triglycerides, **(f)** low-density lipoprotein cholesterol, **(g)** interleukin-1α, **(h)** interleukin-6, and **(i)** interleukin-8 were measured by ELISA. Data are presented as mean ± SD (n = 5). *P < 0.05, **P < 0.01, ***P < 0.001, ****P < 0.0001, ns indicates not significant.

Serum biochemical analysis revealed significant alterations in hormone levels, lipid metabolism markers, and inflammatory cytokines in the androgenetic alopecia model. The model group exhibited substantially elevated testosterone and dihydrotestosterone levels compared to the control group (Figures 4a,b). XZYFD treatment demonstrated dose-dependent reduction of both hormones, with the high-dose group showing the most pronounced effects. The finasteride group achieved the strongest DHT suppression among all treatment groups. Sex hormone-binding globulin levels were significantly reduced in the model group and were partially restored by XZYFD treatment in a dose-dependent manner (Figure 4c).

Lipid metabolism markers demonstrated substantial dysregulation in the model group. Total cholesterol, triglycerides, and low-density lipoprotein cholesterol levels were markedly elevated compared to controls (Figures 4d–f). XZYFD treatment effectively reduced all three lipid parameters across dosage groups, with higher doses showing greater efficacy. The high-dose XZYFD group demonstrated particularly strong effects on triglyceride reduction, achieving levels comparable to or lower than the finasteride group.

Inflammatory cytokine analysis revealed elevated interleukin-1α, interleukin-6, and interleukin-8 levels in the model group (Figures 4g–i). XZYFD treatment significantly suppressed all three inflammatory markers in a dose-dependent manner. The high-dose XZYFD group achieved substantial reductions in inflammatory cytokine levels, though the finasteride group generally demonstrated stronger anti-inflammatory effects. These findings indicated that XZYFD effectively modulated hormone metabolism, lipid homeostasis, and inflammatory responses in androgenetic alopecia mice.

### 3.5 XZYFD suppresses MAPK signaling and lipid metabolism protein expression

**FIGURE 5:**
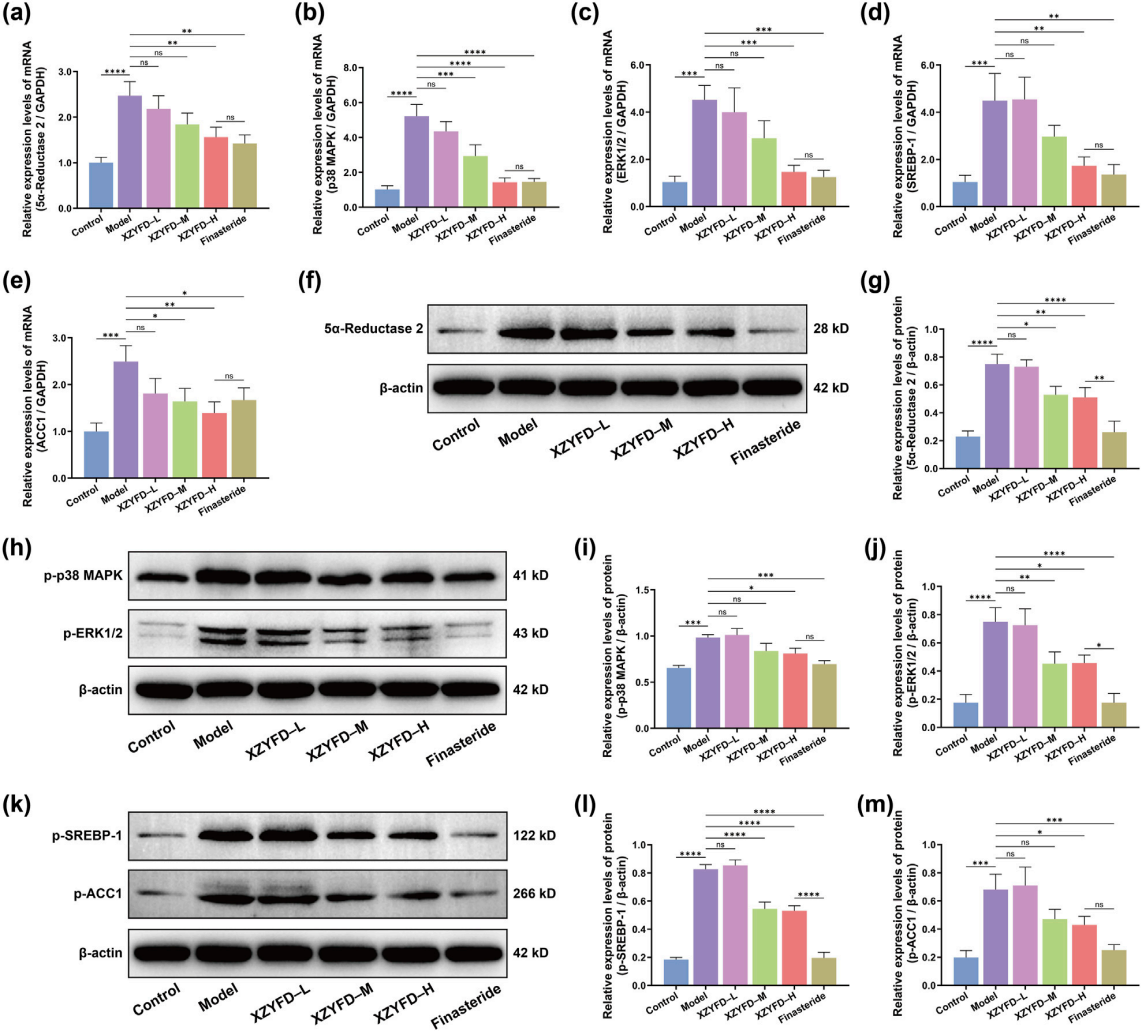
XZYFD regulates gene and protein expression of key targets in androgenetic alopecia mice. **(a–e)** Relative mRNA expression levels of 5α-reductase 2, p38 MAPK, ERK1/2, SREBP-1, and ACC1 determined by quantitative real-time PCR. **(f,g)** Western blot analysis and quantification of 5α-reductase 2 protein expression. **(h–j)** Western blot analysis and quantification of phosphorylated p38 MAPK and phosphorylated ERK1/2 protein expression. **(k–m)** Western blot analysis and quantification of phosphorylated SREBP-1 and phosphorylated ACC1 protein expression. Data are presented as mean ± SD (n = 3). *P < 0.05, **P < 0.01, ***P < 0.001, ****P < 0.0001, ns indicates not significant.

Quantitative real-time PCR analysis revealed significant alterations in target gene expression in the androgenetic alopecia model (Figures 5a–e). The model group exhibited markedly elevated mRNA levels of 5α-reductase 2, p38 MAPK, ERK1/2, SREBP-1, and ACC1 compared to the control group. XZYFD treatment dose-dependently suppressed the expression of all five genes. The high-dose XZYFD group demonstrated the most substantial reductions, achieving levels approaching those observed in the control group. The finasteride group also effectively reduced the expression of these genes, showing comparable or stronger effects than XZYFD treatment depending on the specific target.

Western blot analysis corroborated the gene expression findings at the protein level (Figures 5f–m). The model group displayed significantly increased protein expression of 5α-reductase 2 compared to controls. XZYFD treatment reduced 5α-reductase 2 protein levels in a dose-dependent manner, with the medium-dose and high-dose groups showing pronounced suppression. The finasteride group demonstrated similar efficacy to the high-dose XZYFD group in reducing 5α-reductase 2 expression. Phosphorylated MAPK pathway proteins showed marked elevation in the model group. Both phosphorylated p38 MAPK and phosphorylated ERK1/2 levels were substantially increased compared to controls. XZYFD treatment effectively suppressed the phosphorylation of both proteins across all dosage groups. The high-dose XZYFD group achieved the greatest reduction in MAPK pathway activation, while the finasteride group showed moderate suppressive effects comparable to the low-dose XZYFD group. Lipid metabolism-related protein analysis revealed elevated phosphorylated SREBP-1 and phosphorylated ACC1 levels in the model group. XZYFD treatment dose-dependently reduced the phosphorylation of both proteins. The medium-dose and high-dose XZYFD groups demonstrated substantial suppression of lipid metabolism pathway activation. The finasteride group showed weaker effects on lipid metabolism proteins compared to its effects on 5α-reductase 2 and MAPK signaling proteins.

**FIGURE 6:**
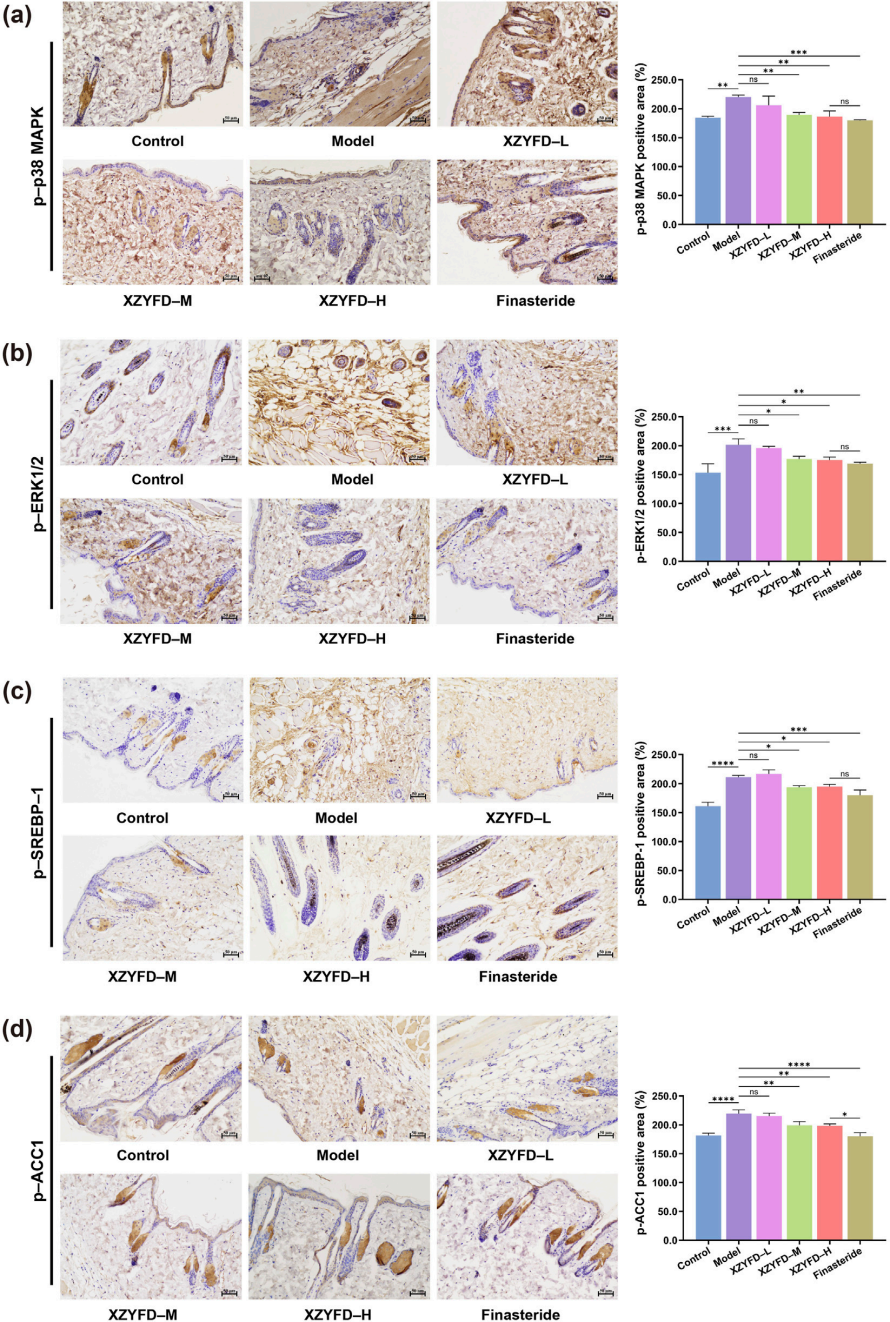
Immunohistochemical analysis of MAPK signaling and lipid metabolism-related proteins in dorsal skin tissues. **(a–d)** Representative immunohistochemical staining images and quantitative analysis of phosphorylated p38 MAPK, phosphorylated ERK1/2, phosphorylated SREBP-1, and phosphorylated ACC1 expression in hair follicles and surrounding dermal tissues. Scale bar represents 50 μm. Data are presented as mean ± SD (n = 5). **P < 0.01, ***P < 0.001, ****P < 0.0001, ns indicates not significant.

Immunohistochemical analysis confirmed the protein expression patterns observed in Western blot studies (Figures 6a–d). The model group exhibited intense positive staining for phosphorylated p38 MAPK, phosphorylated ERK1/2, phosphorylated SREBP-1, and phosphorylated ACC1 in hair follicles and surrounding dermal tissues. XZYFD treatment progressively reduced the immunoreactivity of all four proteins across dosage groups. The high-dose XZYFD group displayed the weakest staining intensity among treatment groups, with staining patterns approaching those observed in control tissues. The finasteride group showed substantial reduction in MAPK pathway protein staining but demonstrated less pronounced effects on lipid metabolism-related proteins. These findings indicated that XZYFD effectively suppressed both MAPK signaling activation and lipid metabolism pathway upregulation in androgenetic alopecia mice, with dose-dependent efficacy across multiple molecular levels.

## 4 Discussion

This study demonstrates that Xiaozhi Yufa decoction ameliorates androgenetic alopecia through coordinated inhibition of MAPK signaling and regulation of lipid metabolism, alongside modulation of androgen metabolism. The integration of network pharmacology with experimental validation confirms that XZYFD acts through multiple pathways simultaneously, providing mechanistic insights into how Traditional Chinese Medicine formulations address complex pathological conditions.

The dose-dependent hair regrowth and follicular restoration observed with XZYFD treatment reveal important implications for understanding therapeutic mechanisms in androgenetic alopecia. The histological improvements extend beyond simple follicular enlargement to encompass restoration of the entire follicular microenvironment, including reduction of pathological subcutaneous adipose accumulation. This suggests that effective treatment requires not only blocking androgenic damage but also actively reversing the metabolic and structural abnormalities that perpetuate follicular dysfunction (Pozo-Pérez et al., 2024; Yan et al., 2024). Previous studies have established that miniaturized follicles retain regenerative capacity if the pathological microenvironment can be corrected (Charoensuksira et al., 2024; Jin et al., 2024; Liu et al., 2025a). This explains why XZYFD’s multi-target approach produces observable effects within 2 weeks. The comparable efficacy to finasteride, particularly at higher doses, indicates that modulating multiple pathways may achieve therapeutic outcomes similar to selective 5α-reductase inhibition, potentially with different safety profiles given the involvement of metabolic regulation rather than solely hormonal suppression.

The suppression of 5α-reductase 2 expression and DHT levels represents a well-established therapeutic mechanism, but the concurrent restoration of SHBG levels provides additional mechanistic significance. SHBG functions beyond simple hormone transport and actively regulates tissue androgen exposure by sequestering testosterone and limiting substrate availability for 5α-reductase conversion (Chen et al., 2023; Dhurat et al., 2020). Studies have shown that SHBG levels inversely correlate with androgenetic alopecia severity (Szybiak-Skora et al., 2025; Vinay et al., 2023). This suggests that SHBG restoration may contribute to reestablishing physiological hormone homeostasis in follicular tissue. This dual mechanism, targeting both enzyme expression and hormone bioavailability, distinguishes XZYFD from direct enzyme inhibitors. It may explain the efficacy of XZYFD despite acting through natural compounds with presumably lower binding affinities than synthetic drugs. The restoration of SHBG also suggests metabolic improvements beyond local effects, as SHBG levels are influenced by insulin sensitivity and hepatic function. This indicates that XZYFD’s effects extend beyond local scalp tissue to address underlying metabolic dysregulation.

The molecular docking studies revealed favorable binding affinities between XZYFD’s active compounds and key target proteins, with caulophyllogenin showing particularly strong interactions with SRD5A2. These computational results provide structural basis for understanding how natural compounds may interact with therapeutically relevant targets (Khantham et al., 2021; Shaikh et al., 2023). The molecular dynamics simulation of the SRD5A2-caulophyllogenin complex provided additional insights into the nature of these interactions. The RMSD fluctuations and varying hydrogen bond numbers observed throughout the 100 ns simulation reflect the dynamic nature of protein-ligand interactions in biological environments. SRD5A2, as a membrane-associated protein, exhibits inherent conformational flexibility that is essential for its catalytic function. The observed conformational changes likely represent the protein sampling different functional states while maintaining ligand engagement. This dynamic behavior is consistent with the induced fit mechanism, where both protein and ligand adjust their conformations to achieve optimal complementarity. The complex maintained binding interactions throughout the simulation period despite these conformational changes, with hydrogen bonds reforming after temporary disruptions. This indicates sufficient interaction stability to support biological activity. These findings align with emerging understanding that natural compounds often achieve therapeutic effects through dynamic, flexible binding modes rather than the rigid, high-affinity interactions characteristic of synthetic drugs (Martin and Frezza, 2022; Salo-Ahen et al., 2021). This is particularly relevant when multiple compounds act synergistically on multiple targets, as occurs with complex herbal formulations.

Androgenetic alopecia represents a distinct pathological entity where metabolic dysfunction directly contributes to hair loss through mechanical and biochemical mechanisms (Cruz et al., 2023; Wu et al., 2023). The excessive subcutaneous adipose accumulation observed in our model reflects dysregulated lipogenesis that extends beyond local fat deposition to indicate broader metabolic disturbance. This adipose expansion compromises follicular blood supply through mechanical compression while creating an altered biochemical environment rich in adipokines and pro-inflammatory mediators. Research has increasingly recognized that androgenetic alopecia involves chronic low-grade inflammation, with elevated cytokines directly damaging follicular stem cells and disrupting normal cycling (Bazmi et al., 2024; Chen et al., 2025). The bidirectional relationship between lipid dysregulation and inflammation creates a self-perpetuating pathological cycle. Lipid mediators activate inflammatory pathways while inflammatory cytokines further disrupt lipid metabolism, establishing a positive feedback loop that sustains follicular damage even after initial androgenic triggers are removed (Kim et al., 2017; Qiu et al., 2022).

The simultaneous suppression of MAPK signaling and SREBP-1-mediated lipogenesis represents a key therapeutic mechanism of XZYFD, as these pathways interact to amplify pathological processes in androgenetic alopecia. MAPK activation serves multiple pathogenic functions. It transduces inflammatory signals, promotes apoptosis in follicular cells, and mediates DHT-induced effects on keratinocytes and dermal papilla cells (Huang et al., 2025; Sintos and Cabrera, 2024). Our findings suggest that MAPK signaling and SREBP-1-mediated lipogenesis may be functionally interconnected in androgenetic alopecia pathogenesis. Studies in other tissues have shown that activated MAPK can phosphorylate and enhance SREBP-1 activity, potentially linking inflammatory signaling to metabolic dysfunction in the follicular microenvironment. This proposed connection may partially explain the frequent association between androgenetic alopecia and metabolic abnormalities including dyslipidemia. Conversely, accumulated evidence from metabolic research suggests that lipid dysregulation can activate MAPK pathways through altered membrane composition and lipid-mediated signaling molecules. If operative in hair follicles, these bidirectional interactions would establish a pathological feedback loop that perpetuates follicular damage. By targeting both pathways simultaneously, XZYFD interrupts this cycle at multiple points rather than addressing only androgenic triggers. This represents a different therapeutic strategy from finasteride, which primarily blocks DHT formation while leaving downstream inflammatory and metabolic pathways intact. The stronger effects of XZYFD on triglyceride levels and lipid metabolism proteins compared to finasteride support this interpretation and suggest particular benefit for patients with metabolic comorbidities. The multi-component nature of XZYFD enables this multi-pathway regulation, with quercetin likely contributing anti-inflammatory and antioxidant effects, flavonoids modulating signaling cascades, and triterpenes providing metabolic regulatory functions (Kim et al., 2020; Su et al., 2025; Zhao et al., 2023). This synergistic action exemplifies how Traditional Chinese Medicine formulations achieve therapeutic effects through network-level modulation rather than single-target inhibition, potentially offering advantages in complex, multifactorial diseases.

Several limitations warrant acknowledgment. The C57BL/6 mouse model, while widely accepted for androgenetic alopecia research, differs from humans in hair cycling patterns and hormonal regulation, which should be considered when extrapolating findings to clinical applications. Our findings derive from this animal model without clinical validation, making conclusions about human therapeutic potential premature. Systematic safety evaluation of the multi-component formulation represents important future work. Comprehensive assessment of potential herb-herb and herb-drug interactions, cumulative toxicity profiles, and adverse effects will be necessary before clinical translation. While individual constituent herbs have documented traditional use, the complete formulation requires thorough safety profiling. The absence of comprehensive chemical profiling limits complete understanding of all bioactive constituents. The qualitative hair growth assessment, though standard in the field, could benefit from complementary quantitative methods in future studies. These limitations indicate that our findings represent an initial mechanistic investigation requiring further validation before clinical application.

## 5 Conclusion

This study establishes that Xiaozhi Yufa decoction effectively ameliorates androgenetic alopecia through coordinated modulation of androgen metabolism, MAPK signaling, and SREBP-1-mediated lipid metabolism. The integration of network pharmacology predictions with comprehensive experimental validation demonstrates that XZYFD acts through multi-target mechanisms rather than single-pathway inhibition, simultaneously suppressing 5α-reductase expression, inhibiting inflammatory MAPK activation, and regulating lipogenic dysregulation. The demonstrated metabolic regulatory effects distinguish XZYFD from conventional androgen-focused treatments and suggest potential therapeutic advantages for patients with metabolic comorbidities. These findings provide mechanistic foundation for clinical investigation of XZYFD as a therapeutic option in androgenetic alopecia, particularly the lipedematous subtype. Future clinical trials will be necessary to establish safety and efficacy in human patients and to determine optimal dosing strategies for clinical application.

## Data Availability

The original contributions presented in the study are included in the article/supplementary material, further inquiries can be directed to the corresponding author.
